# Supplementary material for: The Evolution and Characterization of the RNA Interference Pathways in Lophotrochozoa
Source: Genome Biol Evol. 2024 May 7;16(5):evae098. doi: 10.1093/gbe/evae098 (PMC11114477; doi:10.1093/gbe/evae098)
Supplement: evae098_Supplementary_Data [file evae098_supplementary_data.zip › Supplementary_materials.zip!/selected_miRNAs/NoGe_selected_miRNAs/known_miRNA_families_pdf_files/NMRB01000177.1_Notospermus_geniculatus_strain_Ushimado_scaffold177,_whole_genome_shotgun_sequence_6426.pdf]

Provisional ID	:	NMRB01000177.1_Notospermus_geniculatus_strain_Ushimado_scaffold177,_whole_genome_shotgun_sequence_6426
Score total	:	1320.1
Score for star read(s)	:	3.9
Score for read counts	:	1312.4
Score for mfe	:	0.8
Score for randfold	:	0
Score for cons. seed	:	3
Total read count	:	2586
Mature read count	:	2492
Loop read count	:	0
Star read count	:	94

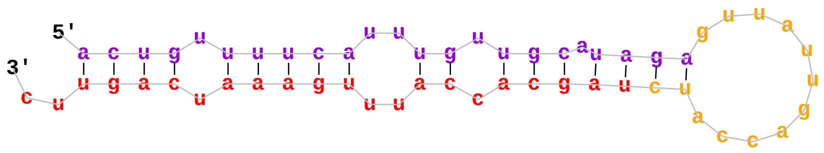

Genome coordinates: NMRB01000177.1 7753-7811

pre-miRNA sequence:
ACUGUUUUCAUUUGUUGCAUAGAACUGUUUUCAUUUGUUGCAUAGAGUUAUUGACCAUCUAGCACCAUUUGAAAUCAGUUC

Mature sequence: UAGCACCAUUUGAAAUCAGUUC

Aligned mature sequence of the *N. geniculatus* pre-miRNA with the top 5 hits from the BLAST search against the pre-miRNA mirGeneDB set:

```
                            ***************** ***
Nge_Mir-29-P2j              uagcaccauuugaaaucaguuc
Gmo-Mir-29-P2b1_3p          uagcaccauuugaaaucgguua
Npo-Mir-29-P2_3p            uagcaccauuugaaaucaguuu
Ebu-Mir-29-P2e_3p           uagcaccauuugaaaucaguua
Lan-Mir-29-P2i_3p           uagcaccauuugaaaucaguuu
Lan-Mir-29-P2j_3p           uagcaccauuugaaaucaguuu
```
